# Supplementary material for: Metabolic pathway inference using multi-label classification with rich pathway features
Source: PLoS Comput Biol. 2020 Oct 1;16(10):e1008174. doi: 10.1371/journal.pcbi.1008174 (PMC7529316; doi:10.1371/journal.pcbi.1008174)
Supplement: S3 Appendix — This file contains additional test results that are not presented in the main article including more in-depth information related to datasets and the ELA robustness metric. (PDF) [file pcbi.1008174.s003.pdf]

Supplementary - Additional Information

Abdur Rahman M. A. Basher, Ryan J. McLaughlin, and Steven J. Hallam

Here, we summarize the characteristics of different datasets used in testing (Section 1). Then, we explain the equalized loss of accuracy metric (Section 2). Finally, additional experimental results, including features analysis (Section 3.1), statistical analysis of pathway prediction algorithms (Section 3.2), pathway prediction results against CAMI data (Section 3.3), and run-time performance of the inference algorithms (Section 3.4). Please consult the primary text for the symbol definitions and the problem formulation.

1 Dataset Characteristics

We developed 12 benchmark datasets used in the experiments, with detailed characteristics summarized in Table Dataset Characteristics. The 12 datasets cover a wide range of cases with diverse multi-label properties, ranging from synthetic to single organism to multiple organisms.

For each dataset $\mathcal{S}$, we use $|\mathcal{S}|$ and $\text{L}(\mathcal{S})$ to represent the number of instances and pathway labels, respectively. In addition, we also present some characteristics of the multi-label datasets, which are denoted as:

1. Label cardinality ($\text{LCard}(\mathcal{S}) = \frac{1}{n}\sum_{i=1}^{i=n}\sum_{j=1}^{j=t}\mathbb{I}[\mathbf{Y}_{i,j} \neq -1]$), where $\mathbb{I}$ is an indicator function. This denotes the average number of pathways in $\mathcal{S}$.
2. Label density ($\text{LDen}(\mathcal{S}) = \frac{LCard(\mathcal{S})}{L(\mathcal{S})}$). This is obtained by dividing $\text{LCard}(\mathcal{S})$ with the number of total pathways in $\mathcal{S}$.
3. Distinct label sets ($\text{DL}(\mathcal{S})$). This notation indicates the number of distinct pathways in $\mathcal{S}$.
4. Proportion of distinct label sets ($\text{PDL}(\mathcal{S}) = \frac{DL(\mathcal{S})}{|\mathcal{S}|}$). This is obtained by dividing DL(.) with the number of instances in $\mathcal{S}$.

The notations $\text{R}(\mathcal{S})$, $\text{RCard}(\mathcal{S})$, $\text{RDen}(\mathcal{S})$, $\text{DR}(\mathcal{S})$, and $\text{PDR}(\mathcal{S})$ represent enzyme reaction level designations as described above for pathways but for the $\mathcal{E}$ in $\mathcal{S}$, and $\text{PLR}(\mathcal{S})$ representing the ratio of $\text{L}(\mathcal{S})$ to $\text{R}(\mathcal{S})$. The experimental multi-label datasets were selected to traverse the genomic information hierarchy encompassing individual, population and community levels of cellular organization and can be compartmentalized based on extent of manual curation and experimental validation. Datasets are ordered by increasing confidence in pathway label information, as: 1)-*golden* (Section 1.1), 2)- symbiont data (Section 1.2), 3)-*CAMI* low complexity data (Section 1.3), 4)- HOTS dataset (Section 1.4), and 5)- *synthetic* datasets (Section 1.5). The preprocessed experimental datasets can be obtained from zenodo.org/record/3821137#.XzBSeXVKjeR

1.1 Golden Dataset

The golden dataset can be decomposed into two tiers following the structure of BioCyc. The T1 golden dataset consisted of six PGDBs retrieved from biocyc website: *EcoCyc (v21)*, *HumanCyc (v19.5)*, *AraCyc (v18.5)*, *YeastCyc (v19.5)*, *LeishCyc (v19.5)*, and *TrypanoCyc (v18.5)*. They were refined to include only those pathways that intersect with the *MetaCyc* database v21 [1]. For each database, we extracted both the enzymatic reactions and the associated pathways.

A composite golden dataset, referred to as *SixDB*, consisted of 63 permuted combinations of T1 PGDBs constructed using the following formula:

$$|\mathcal{S}| = \sum_{k=1}^{k=G}\binom{G}{k} \tag{1}$$

where $|.|$ denotes the number of samples in $\mathcal{S}$ and G is the number of databases, which is 6. Although the biological context of this data was excluded, the pathways were retained.

To better resolve the pathway set difference among the six datasets, we used UpSet [2, 3]. Figure Golden Dataset summarizes the results where the columns of the matrix use binary circled-shaped patterns to define the applied intersected datasets, and the bars, just above the matrix columns, represent the number of elements in each intersection. The bars at the bottom left, plotted along the rows of the matrix, provide information regarding the total intersection size of a dataset Figure Golden Dataset. LeishCyc contained the lowest unique

Table A. Experimental dataset properties The notations $|\mathcal{S}|$, L($\mathcal{S}$), LCard($\mathcal{S}$), LDen($\mathcal{S}$), DL($\mathcal{S}$), and PDL($\mathcal{S}$) represent number of instances, number of pathway labels, pathway labels cardinality, pathway labels density, distinct pathway labels set, and proportion of distinct pathway labels set for $\mathcal{S}$, respectively. The notations R($\mathcal{S}$), RCard($\mathcal{S}$), RDen($\mathcal{S}$), DR($\mathcal{S}$), and PDR($\mathcal{S}$) have similar meanings as before but for the enzymatic reactions $\mathcal{E}$ in $\mathcal{S}$. PLR($\mathcal{S}$) represents a ratio of L($\mathcal{S}$) to R($\mathcal{S}$). The last column denotes the domain of $\mathcal{S}$.

Dataset	$\lvert\mathcal{S}\rvert$	L($\mathcal{S}$)	LCard($\mathcal{S}$)	LDen($\mathcal{S}$)	DL($\mathcal{S}$)	PDL($\mathcal{S}$)	R($\mathcal{S}$)	RCard($\mathcal{S}$)	RDen($\mathcal{S}$)	DR($\mathcal{S}$)	PDR($\mathcal{S}$)	PLR($\mathcal{S}$)	Domain
EcoCyc	1	307	307	1	307	307	1134	1134	1	719	719	0.2707	Escherichia coli K-12 substr.MG1655
HumanCyc	1	279	279	1	279	279	1177	1177	1	693	693	0.2370	Homo sapiens
AraCyc	1	510	510	1	510	510	2182	2182	1	1034	1034	0.2337	Arabidopsis thaliana
YeastCyc	1	229	229	1	229	229	966	966	1	544	544	0.2371	Saccharomyces cerevisiae
LeishCyc	1	87	87	1	87	87	363	363	1	292	292	0.2397	Leishmania major Friedlin
TrypanoCyc	1	175	175	1	175	175	743	743	1	512	512	0.2355	Trypanosoma brucei
SixDB	63	37295	591.9841	0.0159	944	14.9841	210080	3334.6032	0.0159	1709	27.1270	0.1775	Composed from six databases
Symbiont	3	119	39.6667	0.3333	59	19.6667	304	101.3333	0.3333	130	43.3333	0.3914	Composed of Moranella and Tremblaya
CAMI	40	6261	156.5250	0.0250	674	16.8500	14269	356.7250	0.0250	1083	27.0750	0.4388	Simulated microbiomes of low complexity
HOT	4	2178	311.1429	0.1429	781	111.5714	182675	26096.4286	0.1429	1442	206.0000	0.0119	Metagenomic Hawaii Ocean Time-series (10m, 75m, 110m, and 500m)
Synset-1	15000	6801364	453.4243	0.00007	2526	0.1684	30901554	2060.1036	0.00007	3650	0.2433	0.2201	Synthetically generated (uncorrupted)
Synset-2	15000	6806262	453.7508	0.00007	2526	0.1684	34006386	2267.0924	0.00007	3650	0.2433	0.2001	Synthetically generated (corrupted)

information content with 4 distinct pathways and 87 aggregate pathways intersecting other organismal genomes in the T1 golden dataset (Table Dataset Characteristics). In contrast AraCyc data has the highest number in both categories (271 distinct pathways and 510 aggregated pathways).

1.2 Symbiont Dataset

The symbiont dataset represents a nested bacterial symbiosis in the mealybug *Planococcus citri* consisting of *Candidatus Moranella endobia* (GenBank NC-015735) living inside *Candidatus Tremblaya princeps* (GenBank NC-015736) [4]. MetaPathways v2.5 and Pathway Tools version 21 were used to generate ePGDBs with the default settings. The symbiotic *Candidatus Moranella endobia* and *Candidatus Tremblaya princeps* genomes can be downloaded from GenBank under accession numbers NC-015735 and NC-015736).

1.3 CAMI Dataset

The CAMI (Critical Assessment of Metagenome Interpretation) low complexity dataset [5] is a simulated dataset from 40 low complexity genomes. The dataset has various purposes related to evaluating the performance of assembly, profiling, and binning applications. This dataset is placed in a lower order of purity than the previous golden samples because it constitutes a synthetic mock community of microbiomes. MetaPathways v2.5 and Pathway Tools version 21 were used to generate ePGDBs with the default settings. The simulated CAMI low complexity dataset can be obtained from edwards.sdsu.edu/research/cami-challenge-datasets/.

1.4 HOTS Dataset

The the Hawaii Ocean Time-series (HOTS) dataset is composed of complex microbial communities from 25m, 75m, 110m (sunlit) and 500m (dark) ocean depth intervals [6]. Unassembled whole genome shotgun DNA pyrosequences from HOTS (10m, 75m, 110m, and 500m) can be obtained from the NCBI Sequence Read Archive under accession numbers SRX007372, SRX007369, SRX007370, SRX007371. MetaPathways v2.5 and Pathway Tools version 21 were used to generate ePGDBs with the default settings.

1.5 Synthetic Samples Generation

The *in silico* synthetic dataset was constructed by selecting a list of pathways and then creating instances to curate a dataset. This dataset is used to train and evaluate mlLGPR's predictive performance. The data generation process can be summarized in three phases:

Figure A. Matrix layout for all possible intersections among EcoCyc, HumanCyc, AraCyc, YeastCyc, LeishCyc, and TrypanoCyc dataset. Brown circles in the matrix indicate sets that are part of the intersection and their distributions are shown as a vertical bar above the matrix while the aggregated number of pathways from intersected sets for each sample is represented by a horizontal bar at the bottom left.

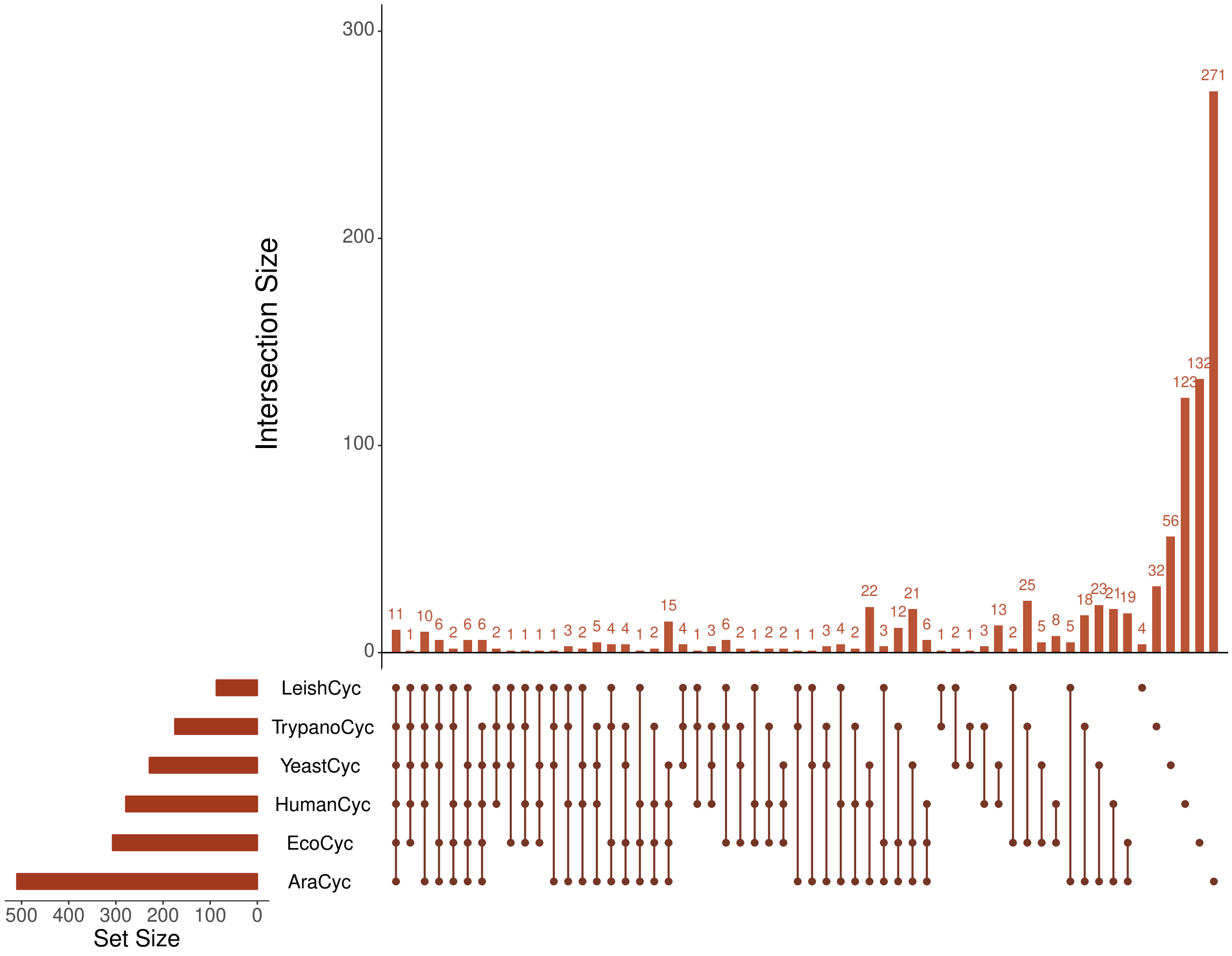

- **Phase 1: Specifying Pathways.** All available pathways from the MetaCyc database and T1 data are collected. A list of pre-specified pathways is selected while truncating the rest. The selected pathway list $\widehat{\mathcal{Y}}$ is used for training and performance evaluation.

- **Phase 2: Generation Process.** We construct an instance by randomly selecting a subset of pathways from $\mathcal{Y}$, i.e., $\widehat{\mathcal{Y}}_i \subset \mathcal{Y}$. Given $\widehat{\mathcal{Y}}_i$, we perform mapping onto MetaCyc to retrieve a list of enzymatic reactions with abundances so as to generate an instance $\mathbf{x}^{(i)}$. Together $(\mathbf{x}^{(i)}, \mathbf{y}^{(i)})$ forms a synthetic sample. Replicating this process n times results in a dataset $\mathcal{S} = \{(\mathbf{x}^{(i)}, \mathbf{y}^{(i)}) | 1 < i \leq n\}$. The enzymatic reactions are indicated by the EC (Enzyme Commission) numbers, which denote the numerical classification of enzymes based on the reactions they catalyze. In the experiment, we consider all EC numbers, including the incomplete ones, such as EC 1.2.3.-.

- **Phase 3: Corruption Process.** The corruption is explicitly applied by first selecting a sample $(\mathbf{x}^{(i)}, \mathbf{y}^{(i)})$, uniformly, from a newly created $\mathcal{S}$. Then, for each pathway $y_j \in \mathbf{y}^{(i)}$, one of the three options is selected: i)- retain y_j, ii)- remove a list of enzymatic reactions associated with y_j, or iii)- insert a list of false enzymatic reactions to y_j. This process is replicated for each individual pathway and for every sample in $\mathcal{S}$ with four specific constraints (reflecting the rules definitions in PathoLogic [7]):

  1. If only a single enzymatic reaction is attached to y_j, we retain that pathway.
  2. If a set of enzymatic reactions is unique to y_j, we do not remove those unique reactions.
  3. If y_j is a biosynthesis pathway, we do not remove the last two enzymatic reactions from that pathway.
  4. If y_j is a biodegradation pathway, we do not remove the first two enzymatic reactions from y_j.

Because the set of pathways, as defined in the MetaCyc database, is unique, distinct, and reflects only a subset of the earth's still unexplored organismal diversity, the *pathway corruption* technique is adopted to create various forms of true pathways that might be encountered in the experimental data due to the errors propagated from the upstream data analysis. In creating the synthetic dataset, the above procedure neglects completely the true biological rules; nonetheless, this dataset will provide a separate unbiased measurement on the performance of mlLGPR. We created two synthetic datasets: *Synset-1* that follows Phase 1 and 2 of the generation process while the *Synset-2* includes Phase 3. The number of expected pathways for both datasets is assumed to follow the Poisson distribution with mean value equal to 500, aligning with the previous work [8].

2 ELA metric

To evaluate the effects of noise on the robustness of mlLGPR performance we employed the *equalized loss of accuracy (ELA)* metric based on the work of Saez and colleagues [9] to describe the expected behavior of a model against noise. This can be expressed as:

$$\begin{aligned} \text{ELA}_\rho &= \text{RLA}_\rho + s(M_0) \\ \text{where } \text{RLA}_\rho &= \frac{M_0 - M_\rho}{M_0} \text{ and } s(M_0) = \frac{1 - M_0}{M_0} \end{aligned} \tag{2}$$

The ELA metric combines both concepts: i)- the robustness of a model, computed by RLA_ρ at a controlled noise threshold ρ and ii)- the performance of a model without noise, i.e., $s(M_0)$, where 1 represents the base accuracy.

3 Experiments

In this section, we demonstrate the performance of mlLGPR on several experiments, extending the works in the main text. These include feature analysis, statistical results of pathway inference algorithms, pathway prediction outputs on CAMI data, and run-time performance of all algorithms.

3.1 Analysis of Features

Features engineering for each example in a genomic dataset can be considered as a transformation process from a raw vector, encoding enzymatic reactions, to a high-dimensional representation of data, incorporating a large number of traits that may be well established to the domain experts, but, the relevance of these features to a given example are often left with little/no information. To properly capture and interpret patterns from a genomic dataset, we introduced many candidate features. Our expectation is that only a handful set of features can characterize a specific target pathway while the majority of candidate features may be irrelevant or redundant that do not contribute in predicting a target pathway. Since mlLGPR incorporates logistic regression with regularization that has built-in feature selection property, we can attain a small subset of candidate relevant features using regression coefficients associated with these features, where a higher regression value entails strong relevancy of the associated feature to a target pathway.

In what follows, we apply a group-based features analysis using the Synset-2 training set to run a series of ablation experiments, in a reverse manner, starting by reaction abundance features, and then incrementally aggregating additional feature set while recording the overall predictive performance of mlLGPR-EN on a suite of 7 T1 golden data. We use the same configurations and settings as described in the main manuscript for all parameters in mlLGPR-EN. Note that the group-based features study is a tractable approach as opposed to the individual feature investigation that is practically prohibitive for the multi-label learning. The results of features ablation experiments are outlined in Table Experiments.

3.1.1 Enzymatic Reaction Abundance Features (AB)

This is the most fundamental and straightforward feature set covering enzymatic reactions with their abundances. Table Experiments indicates that by incorporating this feature set, mlLGPR achieves the highest average recall on EcoCyc with a score of 0.9511 and a comparable F1-score of 0.6952. Figure Enzymatic Reaction Abundance Features (AB) shows that this feature set (50 randomly picked) exhibit non-uniform representations across 100 randomly selected pathways, where a darker entry entails a strong relevancy of the associated feature to a pathway. We extend this experiment to explore features relevancy by arbitrarily picking 5 pathways with their top 5 AB features according to regression values.

From Table Enzymatic Reaction Abundance Features (AB), the regression scores for relevant ECs to each pathway usually tend to be high. For example, the relevant ECs for the following pathways have high scores:

Table B. Ablation tests of mlLGPR-EN trained using Synset-2 on T1 golden datasets. AB: abundance features, RE: reaction evidence features, PP: possible pathway features, PE: pathway evidence features, and PC: pathway common features. mlLGPR is trained using a combination of features, represented by mlLGPR-*, on Synset-2 training set. For each performance metric, '↓' indicates the lower score is better while '↑' indicates the higher score is better.

Methods	Hamming Loss ↓						
	EcoCyc	HumanCyc	AraCyc	YeastCyc	LeishCyc	TrypanoCyc	SixDB
mlLGPR+AB	0.1013	0.0887	0.1025	0.0907	0.1124	0.1073	0.1412
mlLGPR+AB+RE	**0.0788**	0.0697	0.1101	**0.0558**	0.0447	**0.0598**	0.1348
mlLGPR+AB+PP	0.2835	0.2922	0.2898	0.2724	0.2553	0.2759	0.2842
mlLGPR+AB+PE	0.1017	0.0835	**0.1002**	0.0891	0.1172	0.1089	0.1387
mlLGPR+AB+PC	0.1041	0.0938	0.1409	0.0879	0.1081	0.0899	0.1844
mlLGPR+AB+RE+PP	0.2815	0.2882	0.2961	0.2648	0.2526	0.2759	0.2825
mlLGPR+AB+RE+PE	0.0804	**0.0633**	0.1069	0.0550	**0.0380**	0.0590	**0.1281**
mlLGPR+AB+RE+PC	0.0966	0.0732	0.1394	0.0677	0.0515	0.0625	0.1793
mlLGPR+AB+PE+PC	0.1029	0.0899	0.1441	0.0914	0.1148	0.0903	0.1820
mlLGPR+AB+RE+PE+PP	0.2019	0.2070	0.2142	0.1876	0.1884	0.1880	0.2299
mlLGPR+AB+RE+PE+PP	0.2894	0.2993	0.2953	0.2736	0.2530	0.2755	0.2838
mlLGPR+AB+RE+PE+PC	0.0954	0.0816	0.1441	0.0673	0.0451	0.0641	0.1806
mlLGPR+AB+RE+PE+PP+PC	0.2003	0.2063	0.2209	0.1924	0.1924	0.1928	0.2317

Methods	Average Precision Score ↑						
	EcoCyc	HumanCyc	AraCyc	YeastCyc	LeishCyc	TrypanoCyc	SixDB
mlLGPR+AB	0.5478	0.5610	0.7390	0.5000	0.2316	0.3873	0.7323
mlLGPR+AB+RE	**0.6205**	0.6373	0.7275	**0.6410**	0.4293	**0.5414**	0.7412
mlLGPR+AB+PP	0.2755	0.2508	0.3926	0.2303	0.1037	0.1855	0.4300
mlLGPR+AB+PE	0.5473	0.5773	0.7495	0.5048	0.2257	0.3843	0.7402
mlLGPR+AB+PC	0.5618	0.5673	0.7810	0.5113	0.2265	0.4217	0.7650
mlLGPR+AB+RE+PP	0.2795	0.2536	0.3845	0.2375	0.1081	0.1885	0.4322
mlLGPR+AB+RE+PE	0.6187	0.6686	0.7372	0.6480	**0.4731**	0.5455	0.7561
mlLGPR+AB+RE+PC	0.6019	**0.6926**	**0.7992**	0.6330	0.3862	0.5362	**0.7761**
mlLGPR+AB+PE+PC	0.5681	0.5844	0.7645	0.4969	0.2188	0.4223	0.7727
mlLGPR+AB+RE+PE+PP	0.3241	0.3000	0.4730	0.2761	0.1309	0.2283	0.5122
mlLGPR+AB+RE+PE+PP	0.2706	0.2482	0.3870	0.2301	0.1068	0.1873	0.4309
mlLGPR+AB+RE+PE+PC	0.6065	0.6466	0.7744	0.6277	0.4237	0.5291	0.7715
mlLGPR+AB+RE+PE+PP+PC	0.3299	0.2997	0.4580	0.2701	0.1285	0.2244	0.5084

Methods	Average Recall Score ↑						
	EcoCyc	HumanCyc	AraCyc	YeastCyc	LeishCyc	TrypanoCyc	SixDB
mlLGPR+AB	**0.9511**	0.9068	0.7608	**0.9258**	0.9770	0.9429	0.6775
mlLGPR+AB+RE	0.9055	0.8566	0.7275	0.8734	0.9080	0.8971	0.6774
mlLGPR+AB+PP	0.8176	0.8280	**0.7961**	0.8559	0.8391	0.8800	0.7696
mlLGPR+AB+PE	0.9414	**0.9104**	0.7569	0.9170	**0.9885**	**0.9486**	0.6795
mlLGPR+AB+PC	0.6515	0.6344	0.4196	0.6900	0.8851	0.8000	0.3827
mlLGPR+AB+RE+PP	0.8339	0.8280	0.7765	0.8690	0.8736	0.9029	**0.7768**
mlLGPR+AB+RE+PE	0.8827	0.8459	0.7314	0.8603	0.9080	0.8914	0.6904
mlLGPR+AB+RE+PC	0.6059	0.6057	0.4137	0.6026	0.8391	0.7200	0.3820
mlLGPR+AB+PE+PC	0.6384	0.6452	0.4137	0.6900	0.9080	0.8229	0.3923
mlLGPR+AB+PP+PC	0.6091	0.6559	0.5333	0.6594	0.7931	0.7200	0.5053
mlLGPR+AB+RE+PE+PP	0.8143	0.8423	0.7922	0.8603	0.8621	0.8914	0.7758
mlLGPR+AB+RE+PE+PC	0.6124	0.5771	0.4039	0.6332	0.8621	0.6743	0.3776
mlLGPR+AB+RE+PE+PP+PC	0.6287	0.6487	0.5137	0.6594	0.7931	0.7257	0.5074

Methods	Average F1 Score ↑						
	EcoCyc	HumanCyc	AraCyc	YeastCyc	LeishCyc	TrypanoCyc	SixDB
mlLGPR+AB	0.6952	0.6932	0.7498	0.6493	0.3744	0.5491	0.6754
mlLGPR+AB+RE	**0.7364**	0.7309	0.7275	**0.7394**	0.5830	0.6753	0.6938
mlLGPR+AB+PP	0.4122	0.3850	0.5259	0.3630	0.1846	0.3065	0.5386
mlLGPR+AB+PE	0.6922	0.7065	**0.7532**	0.6512	0.3675	0.5470	0.6802
mlLGPR+AB+PC	0.6033	0.5990	0.5459	0.5874	0.3607	0.5523	0.4683
mlLGPR+AB+RE+PP	0.4186	0.3882	0.5143	0.3730	0.1924	0.3119	0.5422
mlLGPR+AB+RE+PE	0.7275	**0.7468**	0.7343	0.7392	**0.6220**	**0.6768**	**0.7098**
mlLGPR+AB+RE+PC	0.6039	0.6463	0.5452	0.6174	0.5290	0.6146	0.4853
mlLGPR+AB+PE+PC	0.6012	0.6133	0.5369	0.5777	0.3527	0.5581	0.4779
mlLGPR+AB+PP+PC	0.4231	0.4117	0.5014	0.3892	0.2248	0.3466	0.4857
mlLGPR+AB+RE+PE+PP	0.4062	0.3834	0.5199	0.3631	0.1901	0.3095	0.5407
mlLGPR+AB+RE+PE+PC	0.6094	0.6098	0.5309	0.6304	0.5682	0.5930	0.4805
mlLGPR+AB+RE+PE+PP+PC	0.4327	0.4100	0.4843	0.3832	0.2212	0.3428	0.4847

Figure B. Heatmap representing regression values for 50 randomly selected enzymatic reaction abundance features with their associated 100 randomly selected pathways. The entries is color-coded on a gradient scale within $[-1, 1]$ interval, where a higher intensity entry entails a higher coefficient score, and vice versa. The horizontal axis indicates the indices of pathways, while the vertical axis represents the indices of features.

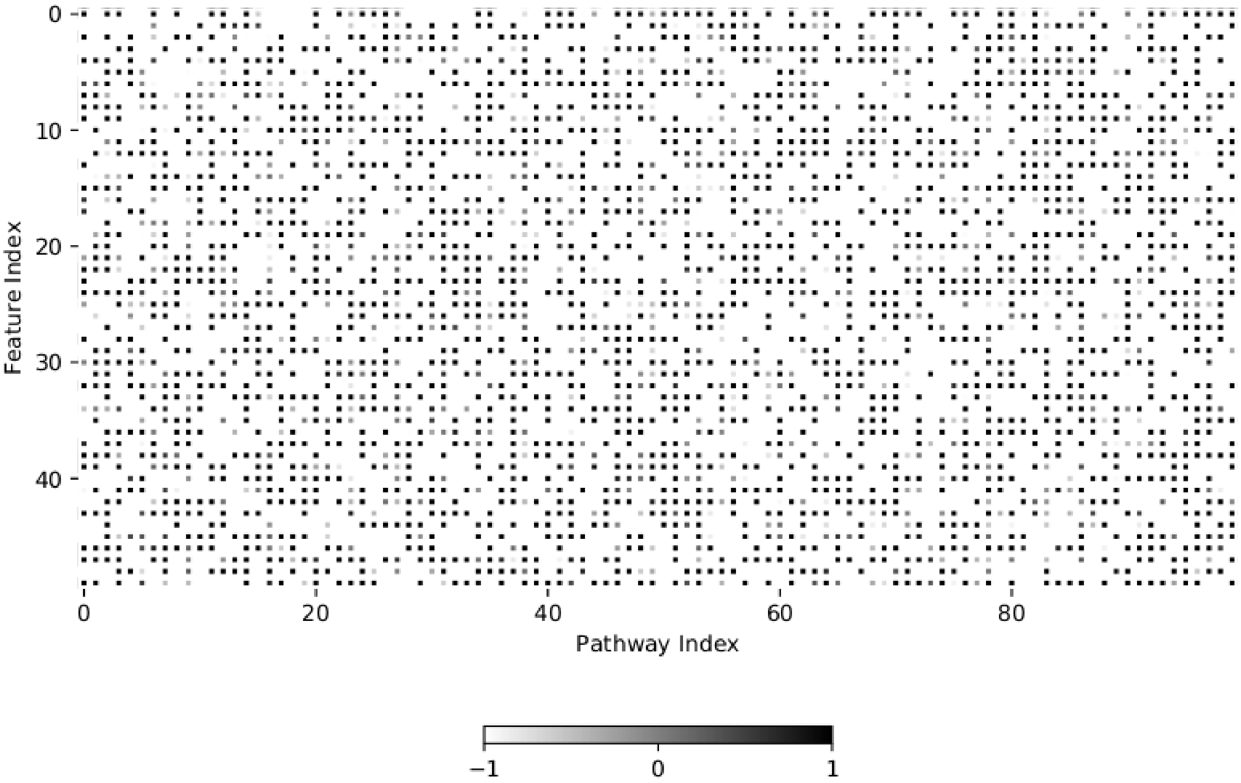

Table C. Arbitrarily selected 5 pathways with their top 5 features according to coefficient values. For each metric, ↑ indicates the higher score is better. Note, the underlined text represents irrelevant features.

MetaCyc Pathway	#	EC	Coefficient ↑
TCA cycle I (prokaryotic)	1	EC-1.1.5.4	371.7255
	2	EC-6.2.1.5	183.7094
	3	EC-1.3.5.1	107.8370
	4	EC-1.1.1.42	81.7021
	5	EC-2.3.3.1	59.3995
pregnenolone biosynthesis	1	EC-1.3.1.29	28.5392
	2	EC-2.8.3.20	22.1299
	3	EC-2.4.2.44	20.9005
	4	EC-5.4.99.58	20.1949
	5	EC-3.5.2.10	20.0073
4,5-dichlorocat-echol degradation	1	EC-3.1.1.45	214.7650
	2	EC-1.13.11.M6	178.4025
	3	EC-5.5.1.7	145.1341
	4	EC-1.14.13.55	32.2769
	5	EC-1.13.11.M3	29.4273
diphthamide biosynthesis I (archaea)	1	EC-2.1.1.98	824.8856
	2	EC-2.5.1.108	548.2827
	3	EC-6.3.1.14	545.2099
	4	EC-2.1.1.239	47.1502
	5	EC-1.11.2	46.1283
D-gluconate degradation	1	EC-2.7.1.12	525.4430
	2	EC-3.2.1.157	32.0726
	3	EC-3.5.99	31.9282
	4	EC-1.1.1.346	31.5193
	5	EC-4.2.1.11	31.2862

Figure C. Heatmap representing regression values for 68 enzymatic reaction evidence features with their associated 100 randomly selected pathways. The entries is color-coded on a gradient scale within $[-1, 1]$ interval, where a higher intensity entry entails a higher coefficient score, and vice versa. The horizontal axis indicates the indices of pathways, while the vertical axis represents the indices of features.

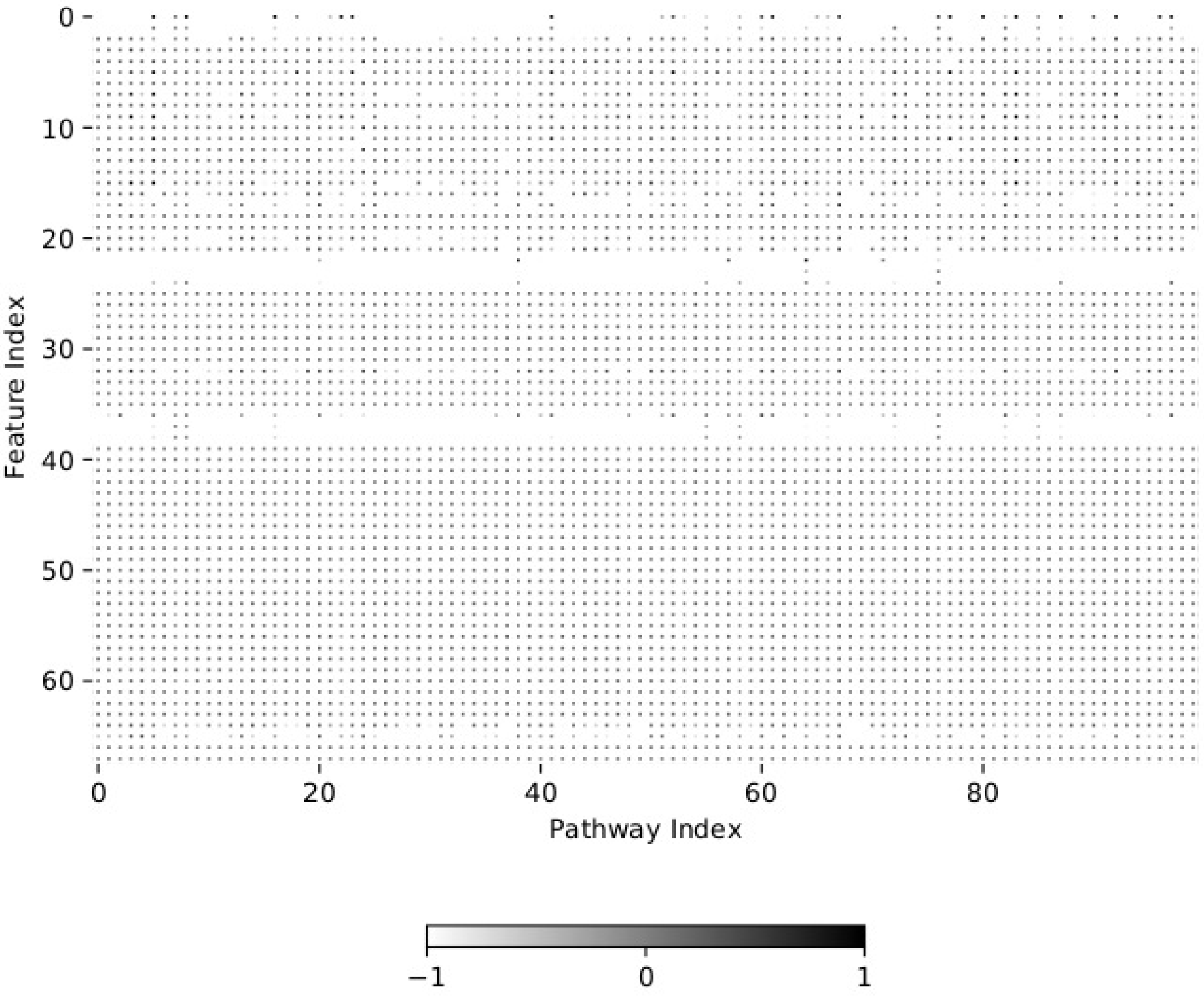

TCA cycle I (prokaryotic), *4,5-dichlorocatechol degradation*, and *diphthamide biosynthesis I (archaea)*. Since ECs were not associated with *pregnenolone biosynthesis* pathway, all the corresponding ECs are considered irreverent.

Based on these results, we confirm that mlLGPR was able to retrieve the top 5 relevant ECs to pathways. Furthermore, the AB feature set is demonstrated to be the most essential to recovering pathways. However, solely relying on this feature set may enforce mlLGPR to neglect pathways that have low number or no enzymatic reactions. A potential solution is to aggregate additional features as examined in the following sections.

3.1.2 Adding Enzymatic Reaction Evidence Features (+RE)

The enzymatic reaction evidence (RE) features describe the properties of enzymatic reactions participating in pathways, as encoded in MetaCyc v21. A total of 68 features were defined. Some RE features test the fraction of ECs present in pathways while other features compute the fraction of some ECs being present in either at the beginning of specific pathways (e.g. biodegradation pathways) or at the rear of some pathways (e.g. biosynthesis pathways). We refer readers to the supplementary file for the full description of this feature set. As shown in Table Experiments, the addition of RE features on top of AB features resulted in substantial improvements of mlLGPR on YeastCyc, LeishCyc, and TrypanoCyc, recording average F1 scores of 0.7394, 0.5830, and 0.6753, respectively. While mlLGPR+AB+RE marginally outperformed mlLGPR+AB on EcoCyc, HumanCyc, and SixDB, its performance on AraCyc data was reduced, resulting in an average F1 score of 0.7275. The coefficients of this feature set are seen to have a uniform representation across pathways as indicated in Figure Adding Enzymatic Reaction Evidence Features (+RE). Moreover, 6 RE features (*fraction-total-possible-pathways-to-distinct-*

Table D. Arbitrarily selected 5 pathways with their top 5 RE features values. For each metric, ↑ indicates the higher score is better.

MetaCyc Pathway	#	Enzymatic Reaction Evidence Feature	Coefficient ↑
TCA cycle I (prokaryotic)	1	fraction-total-ecs-act-as-final-reactions	0.3512
	2	fraction-total-ec-act-in-biosynthesis-pathway	0.3362
	3	fraction-total-ecs-act-as-initial-reactions	0.3016
	4	num-distinct-reactions-present-or-orphaned-in-pathways	0.1983
	5	fraction-ec-contributes-in-subpathway-over-total-pathways	0.1642
pregnenolone biosynthesis	1	fraction-total-ecs-contribute-in-subpathway-as-inside-superpathways	2.6116
	2	fraction-total-ecs-act-as-initial-and-final-reactions	1.6005
	3	has-distinct-ecs-present-in-pathways	0.2216
	4	majority-of-ecs-present-in-pathways	0.2096
	5	fraction-total-distinct-ecs-act-as-initial-reactions	0.1860
4,5-dichlorocat-echol degradation	1	fraction-total-ecs-act-in-deg-or-detox-pathway	0.3034
	2	fraction-total-ecs-to-total-reactions	0.2434
	3	fraction-total-ecs-act-as-initial-and-final-reactions	0.2273
	4	majority-of-ecs-absent-in-pathways	0.2202
	5	fraction-total-ecs-contribute-in-subpathway-as-inside-superpathways	0.1965
diphthamide biosynthesis I (archaea)	1	fraction-total-ecs-act-as-initial-reactions	0.6000
	2	fraction-total-ecs-act-as-final-reactions	0.4972
	3	fraction-total-ec-act-in-biosynthesis-pathway	0.4559
	4	fraction-ec-contributes-in-subpathway-over-total-pathways	0.2704
	5	majority-of-ecs-present-in-pathways	0.2676
D-gluconate degradation	1	fraction-total-ecs-act-as-final-reactions	0.6994
	2	fraction-total-ecs-act-as-initial-reactions	0.6768
	3	fraction-total-ec-contribute-in-unique-reaction	0.5939
	4	fraction-total-ec-act-in-biosynthesis-pathway	0.5577
	5	fraction-total-ecs-to-ecs-mapped-to-single-pathways	0.4574

pathways, *fraction-total-pathways-over-total-ecs*, *fraction-total-pathways-over-distinct-ec*, *fraction-total-distinct-pathways-over-distinct-ec*, *fraction-total-reactions-over-distinct-pathways*, *fraction-distinct-reaction-over-distinct-pathways*) are distinctly associated with 5 pathways (*polyhydroxybutanoate biosynthesis*, *oligomeric urushiol biosynthesis*, *pyruvate fermentation to acetone*, *cob(II)yrinate a,c-diamide biosynthesis I (early cobalt insertion)*, and *dimethylsulfoniopropanoate degradation II (cleavage)*), which are all found to be relevant.

Similar to the previous section, Table Adding Enzymatic Reaction Evidence Features (+RE) shows the 5 selected pathways with their associated top RE features, where mlLGPR+AB+RE was able to retrieve top 5 relevant features associated with these pathways, thereby, supporting our previous observation. For example, the *fraction-total-ec-act-in-biosynthesis-pathway* feature for the *TCA cycle I (prokaryotic)* pathway is ranked third with a score of 0.3362. This feature describes fractions of total ECs contributing to this biosynthetic pathway. For *4,5-dichlorocatechol degradation* pathway, its top 4 features (*fraction-total-ecs-act-in-deg-or-detox-pathway*, *fraction-total-ecs-to-total-reactions*, *fraction-total-ecs-act-as-initial-and-final-reactions*, and *majority-of-ecs-absent-in-pathways*) are all relevant. These results demonstrate that by incorporating RE features on top of AB features the performance of mlLGPR was improved.

3.1.3 Adding Pathway Evidence Features (+PE)

The pathway evidence (PE) features include both categorical and numerical features, which are expected to capture various properties of pathways, as defined in MetaCyc v21. A total of 32 PE features were defined. The description about this feature set is provided in the supplementary file. As before, we train mlLGPR-EN model by incorporating PE features on top of AB features while evaluating the model's performance. From Table Experiments, we exhibit a similar trend as with mlLGPR+AB+RE, except for LeishCyc where the performance of mlLGPR+AB+PE model drops drastically recording an average F1 score of 0.3675 which is similar to mlLGPR+AB. This suggests that PE features are as effective as RE features for some samples while shares the strengths of AB features for other data (see Figure. Adding Pathway Evidence Features (+PE) for non-uniform scores representation of PE features). Moreover, the top 10 PE features span across many pathways, as shown in Table Adding Pathway Evidence Features (+PE). In summary, the PE feature set shares similar behaviors as RE features, implying fusing AB, RE, and PE features may escalate pathway prediction performance as discussed in Section Gradually Aggregating All Possible Combinations of Previous Features.

Figure D. Heatmap representing regression values for 32 pathway evidence features with their associated 100 randomly selected pathways. The entries is color-coded on a gradient scale within $[-1, 1]$ interval, where a higher intensity entry entails a higher coefficient score, and vice versa. The horizontal axis indicates the indices of pathways, while the vertical axis represents the indices of features.

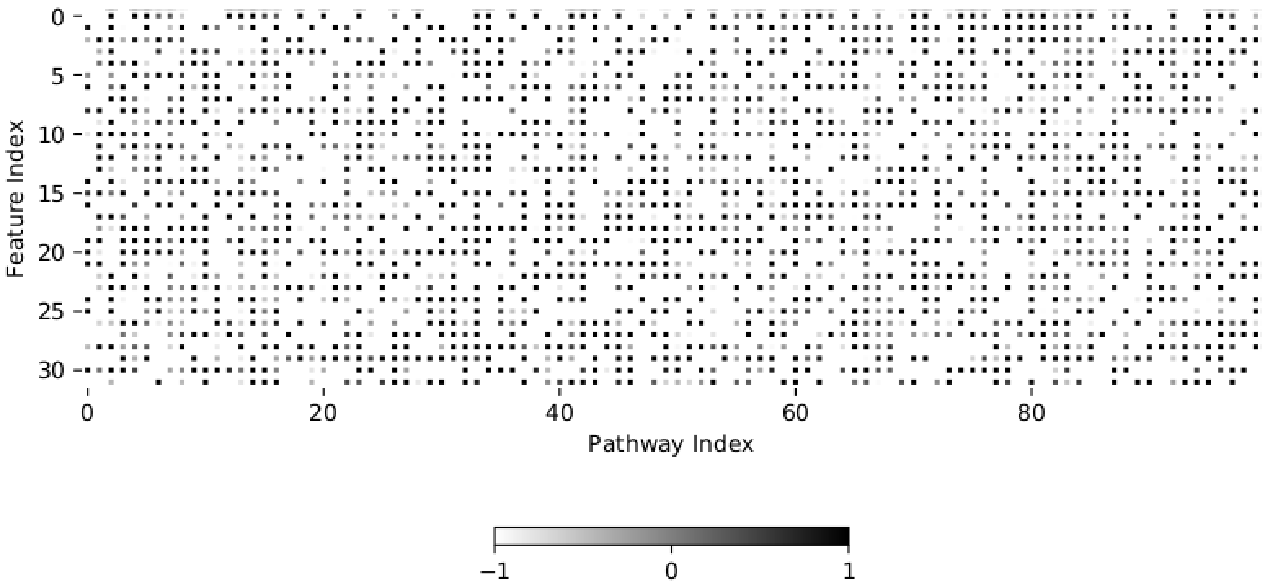

Table E. Most common 10 PE features shared among 2526 pathways.

Pathway Evidence Feature	Number of Pathways
majority-of-ecs-absent-in-pathway	851
all-initial-ecs-present-in-deg-or-detox-pathway	832
ecs-mostly-present-in-pathway	828
all-initial-and-final-ecs-present-in-pathway	818
prob-ecs-mostly-present-in-pathway	808
all-ecs-present-in-pathway	807
one-ec-present-but-in-minority-in-pathway	803
has-distinct-ecs-present-in-pathway	800
most-ecs-absent-not-distinct-in-pathway	800
fraction-reactions-present-or-orphaned-distinct-in-pathway	799

3.1.4 Adding Possible Pathway (+PP) and Pathway Commmon (+PC) Features

The possible pathway (PP) features capture the likeliness of pathways being present in samples (conditioned on true mappings of ECs onto pathways) while the pathway common features are designated to recognize (mis-)matches between a list of ECs from samples and the true mappings of pathways to ECs. The purpose here is of twofold: i)- if a set of pathways exceeding a specific threshold (0.5 in this experiment) then these pathways are more likely to occur in a sample and ii)- for any pathway, if its true ECs are found to be present then that pathway may also be present in a given example.

By training mlLGPR-EN with +AB+PP and +AB+PC features, separately, on top of AB features, the two models are observed (Table Experiments) to degrade their overall performances on T1 golden data. For example, both models recorded average F1 scores of 0.3850 (mlLGPR+AB+PP) and 0.5990 (mlLGPR+AB+PC) on HumanCyc sample which are significantly worse than the score obtained for mlLGPR+AB (0.6932). These results indicate that both features are irrelevant to pathways, hence, they do not contribute in learning and neither in prediction.

3.1.5 Gradually Aggregating All Possible Combinations of Previous Features

After individual assessment of each feature set, we study the impact of aggregating all possible combinations of features sets on mlLGPR-EN's performances. The objective of this experiment is obtain a minimum subset of feature categorizes that will result in the most predictive gain of mlLGPR on T1 golden datasets. From Table Experiments, we observe that mlLGPR+AB+RE+PE achieves the best overall performances on all metrics.

Figure E. Comparison of seven methods against each other with the Nemenyi test using CD diagrams. Groups of methods that are not significantly different (at $\tau = 0.05$) are connected. (a)- CD diagram for Hamming loss. (b)- CD diagram for average precision score. (c)- CD diagram for average recall score.(d)- CD diagram for average F1 score.

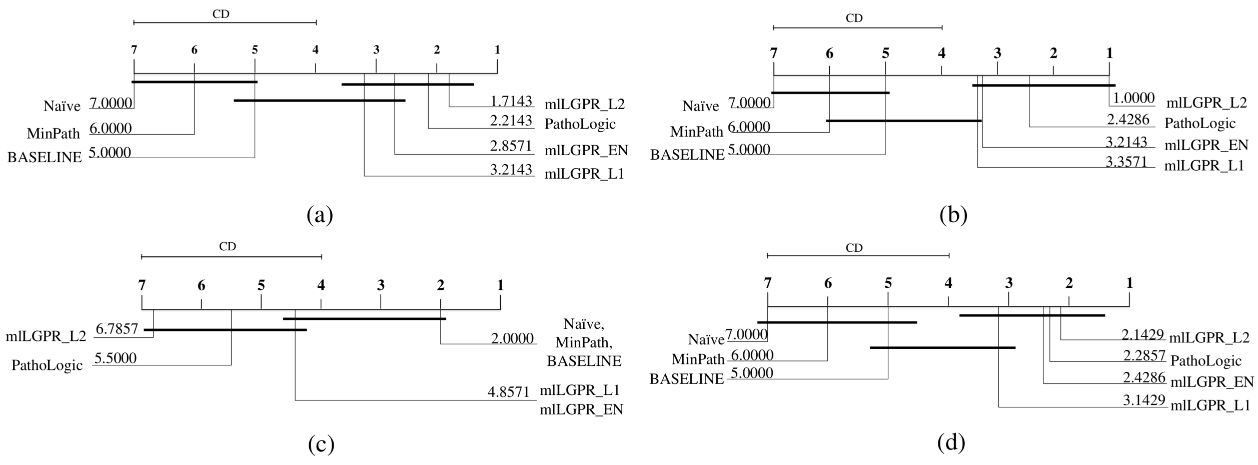

In particular, mlLGPR+AB+RE+PE ranks first with regard to average F1 scores on HumanCyc (0.7468), LeishCyc(0.6220), TrypanoCyc (0.6768), and SixDB (0.7078). Therfore, we recommend this composition of features in the pathway prediction task.

3.2 Statistical Analyses of Pathway Prediction Algorithms

mlLGPR performance was compared to four additional prediction methods including BASELINE, Naïve v1.2 [10], MinPath v1.2 [10] and PathoLogic v21 [7] and the results of pathway prediction were compared and ranked using the *Friedman test* [11]. Let r_i^j denote the rank of the m-th of $\mathbf{C}$ algorithms, based on a performance metric discussed in the main manuscript, on the i-th of $|\mathcal{S}|$ dataset. Also, let $R_m = \frac{1}{|\mathcal{S}|}\sum_i r_i^m$ be the average rank for the m-th algorithm under the null-hypothesis that states "all algorithms are equally likely to perform". Then, the Friedman statistic is distributed according to the F-distribution with $\mathbf{C} - 1$ and $(\mathbf{C} - 1)(|\mathcal{S}| - 1)$ degrees of freedom:

$$F_F = \frac{(|\mathcal{S}| - 1)\chi_F^2}{|\mathcal{S}|(\mathbf{C} - 1) - \chi_F^2}$$
$$\text{where } \chi_F^2 = \frac{12|\mathcal{S}|}{\mathbf{C}(\mathbf{C}+1)}[\sum_m R_m^2 - \frac{\mathbf{C}(\mathbf{C}+1)^2}{4}] \tag{3}$$

The results of this test are summarized in Table Statistical Analyses of Pathway Prediction Algorithms. With 7 algorithms and 7 datasets, the critical value of $F_F(6, 36)$ at significance level $\tau = 0.05$ is 2.3638, so we reject the null-hypothesis in terms of all metrics because their F_F values are higher than the critical value.

Table F. Summary of the Friedman statistics F_F for 7 algorithms and 7 datasets. The critical value τ is set to 0.05 significance level.

Metric	F_F	Critical value ($\tau = 0.05$)
Hamming Loss	41.4783	
Average Precision	111.3000	2.3638
Average Recall	57.5250	
Average F1	32.1111	

Consequently, we proceed with a *Nemenyi (post-hoc)* [11] test to analyze the relative performance among the pathway prediction algorithms where the mlLGPR-EN is treated as the control algorithm:

$$\text{Critical Difference (CD)} = q_\tau \sqrt{\frac{\mathbf{C}(\mathbf{C}+1)}{6|\mathcal{S}|}} \tag{4}$$

where $q_\tau = 2.949$ at significance level $\tau = 0.05$, hence, the critical difference (CD) = 3.4052 ($\mathbf{C} = 7, |\mathcal{S}| = 7$) (see the paper [11]). This means the performance of mlLGPR-EN in compare to the remaining pathway inference algorithm is considered to be significantly different if the average ranking based on a performance metric on 7 datasets differs by more than 3.4052 CDs. Figure Statistical Analyses of Pathway Prediction Algorithms shows the CD diagrams for four evaluation metrics at 0.05 significance level, where the average rank of each comparing algorithm is marked along the axis. In each sub-figure, methods that are not considered significantly different are interconnected with a thick line. In summary, among 49 comparisons (7 methods $\times 7$ datasets), all variants of mlLGPR statistically outperformed the other methods in terms of Hamming loss and average F1 metrics. With regard to average precision, all variants of mlLGPR achieve statistically comparable performances with PathoLogic, however, both mlLGPR-EN and mlLGPR-L1 have similar rankings as BASELINE, Naïve, and MinPath in terms of average recall. These observations indicate the competitive performance of mlLGPR-EN, against the rest of the pathway prediction algorithms, in all of the evaluation metrics.

3.3 Pathway Prediction on CAMI data

we evaluated the pathway prediction performance of mlLGPR (using elastic net penalty with reaction and pathway evidence features) on CAMI low complexity data. Table Pathway Prediction on CAMI data shows performance scores for mlLGPR-EN (+AB+RE+PE) on the CAMI dataset. Although recall was high (0.7827) precision and F1 scores were low when compared to the T1 golden datasets.

Table G. Predictive performance of mlLGPR-EN with AB, RE and PE feature sets on CAMI low complexity data.

Metric	mlLGRPR-EN (+AB+RE+PE)
Hamming Loss (↓)	0.0975
Average Precision Score (↑)	0.3570
Average Recall Score (↑)	0.7827
Average F1 Score (↑)	0.4866

3.4 Run-Time Performance of Pathway Prediction Algorithms

In this section, we perform time complexity analysis of the following pathway prediction algorithms: BASELINE, Naïve, MinPath, PathoLogic, and mlLGPR-EN (+AB+RE+PE). We divide our analysis according to: *preprocessing* (including feature engineering), *learning*, and *prediction* time on SixDB data. Since BASELINE, Naïve, MinPath, and PathoLogic do not incorporate learning, their associated time performances were not reported. For PathoLogic, the preprocessing and building features are its intrinsic properties, hence, we only report the inference time. For mlLGPR-EN, we document the learning time based on Synset-2 training set while both preprocessing and prediction time are recorded on SixDB. The experiment was conducted using parameters settings, described in the main manuscript, on a workstation that has a 3.4-GHz Intel CPU processor and 32GB RAM, running MAC-OS version 10 with a single threaded process.

Table H. Run-time performance (mean ± std. deviation in seconds) of each pathway prediction method on SixDB dataset. The (–) symbol means the task is not applicable for the associated method.

Methods	Preprocessing	Learning	Inference	Total
BASELINE	49.2293 ± 0.3076	–	54.8320 ± 1.1618	104.0613 ± 1.4694
Naïve	50.5800 ± 0.3176	–	125.4297 ± 5.7900	176.0097 ± 6.1076
MinPath	50.5800 ± 0.3176	–	125.4297 ± 5.7900	176.0097 ± 6.1076
PathoLogic	–	–	14063.2027 ± 25.1462	14112.4320 ± 25.4538
mlLGPR-EN (+AB+RE+PE)	116.2113 ± 0.6935	51102.8850 ± 297.6997	2.8927 ± 0.0127	51221.9890 ± 298.4060

Table Run-Time Performance of Pathway Prediction Algorithms shows the resulted time performances analysis. BASELINE, MinPath, and Naïve algorithms achieve comparable preprocessing and inference times, yielding $49 - 50$ and $54 - 126$ seconds, respectively. PathoLogic algorithm is observed to have the worst inference time (14063.2027 ± 25.1462), perhaps, it employs an exhaustive search over the biologically defined rules to predict a set of true-positive pathways. For mlLGPR-EN (+AB+RE+PE), the learning is always the slowest task consuming 51102.8850 seconds while the prediction task is considerably fast (~ 2.8927 seconds). Based on these results, mlLGPR has the best overall prediction time in contrast to PathoLogic which has a less optimal prediction time.

References

[1] Caspi R, Billington R, Ferrer L, Foerster H, Fulcher CA, Keseler IM, et al. The MetaCyc database of metabolic pathways and enzymes and the BioCyc collection of pathway/genome databases. Nucleic Acids Research. 2016;44(D1):D471–D480.

[2] Lex A, Gehlenborg N, Strobelt H, Vuillemot R, Pfister H. UpSet: visualization of intersecting sets. IEEE transactions on visualization and computer graphics. 2014;20(12):1983–1992.

[3] Lex A, Gehlenborg N. Points of view: Sets and intersections; 2014.

[4] McCutcheon JP, Von Dohlen CD. An interdependent metabolic patchwork in the nested symbiosis of mealybugs. Current Biology. 2011;21(16):1366–1372.

[5] Sczyrba A, Hofmann P, Belmann P, Koslicki D, Janssen S, Dröge J, et al. Critical assessment of metagenome interpretation—a benchmark of metagenomics software. Nature methods. 2017;14(11):1063.

[6] Stewart FJ, Sharma AK, Bryant JA, Eppley JM, DeLong EF. Community transcriptomics reveals universal patterns of protein sequence conservation in natural microbial communities. Genome biology. 2011;12(3):R26.

[7] Karp PD, Latendresse M, Paley SM, Krummenacker M, Ong QD, Billington R, et al. Pathway Tools version 19.0 update: software for pathway/genome informatics and systems biology. Briefings in bioinformatics. 2016;17(5):877–890.

[8] Shafiei M, Dunn KA, Chipman H, Gu H, Bielawski JP. BiomeNet: A Bayesian model for inference of metabolic divergence among microbial communities. PLoS Comput Biol. 2014;10(11):e1003918.

[9] Sáez JA, Luengo J, Herrera F. Evaluating the Classifier Behavior with Noisy Data Considering Performance and Robustness. Neurocomput. 2016;176(C):26–35.

[10] Ye Y, Doak TG. A parsimony approach to biological pathway reconstruction/inference for genomes and metagenomes. PLoS Comput Biol. 2009;5(8):e1000465.

[11] Demšar J. Statistical comparisons of classifiers over multiple data sets. Journal of Machine learning research. 2006;7(Jan):1–30.
