# Supplementary material for: Metabolic pathway inference using multi-label classification with rich pathway features
Source: PLoS Comput Biol. 2020 Oct 1;16(10):e1008174. doi: 10.1371/journal.pcbi.1008174 (PMC7529316; doi:10.1371/journal.pcbi.1008174)
Supplement: Attachment — Submitted filename: Response2Reviewers.pdf [file pcbi.1008174.s007.pdf]

Response to Reviewer's Comments on "Metabolic pathway inference using multi-label classification with rich pathway features"

Abdur Rahman M. A. Basher, Ryan J. McLaughlin, and Steven J. Hallam

We thank the anonymous reviewers for their thoughtful comments and questions. We carefully evaluated the points raised, and hopefully address them adequately below. **C**: Reviewer's Comment; **R**: Authors' Response.

Reviewer A.

C1 In line 113, the authors wrote that they considered five types of feature vectors based on the work of Dale et al. However, they did not describe the details about whether they used exactly the same features or expanded the features relative to the previous work. The features need to be compared in more detail. Since the previous work also used logistic regression, the authors are strongly encourage to explain what improvements have been made compared to the previous work.

R1 We thank the reviewer for giving us the opportunity to clarify the specific problem covered in the current paper. Dale and colleagues developed multiple machine learning models intended to predict the presence/absence of pathways for individual organismal genomes given pathway properties [1], while in this work the objective is to infer a set of expected true pathways given a list of enzymatic reactions in both organismal and multi-organismal genomes e.g. microbiomes. Each element of data in [1] constitute a triplet format (organism, pathway, presence/absence) whereas in this work it is in a tuple form (enzymatic reactions, subset of pathways). Therefore, the two studies solve different aspects of the metabolic pathway prediction problem. We can visually elaborate on the difference between the two approaches. According to [1] the problem can be represented for two organisms, E. coli and A. thaliana as:

	f_1	f_2	f_3	$\cdots$	f_{123}	is-present?
**E. coli**						
TCA cycle I (prokaryotic)	0	1	10	$\cdots$	1	1
glycine biosynthesis III	1	0	0	$\cdots$	0	0
L-asparagine biosynthesis I	1	0	1	$\cdots$	1	1
L-homocysteine biosynthesis	1	0	0	$\cdots$	1	0
$\vdots$	$\vdots$	$\vdots$	$\vdots$	$\vdots$	$\vdots$	$\vdots$
**A. thaliana**						
TCA cycle I (prokaryotic)	0	1	2	$\cdots$	0	0
glycine biosynthesis III	1	0	1	$\cdots$	1	1
L-asparagine biosynthesis I	1	0	1	$\cdots$	1	1
L-homocysteine biosynthesis	1	0	2	$\cdots$	1	1
$\vdots$	$\vdots$	$\vdots$	$\vdots$	$\vdots$	$\vdots$	$\vdots$

where each row in the left matrix corresponds to an instance of pathways consisting of 123 column features extracted from the corresponding pathways. For example, the feature f_1 may encode `biosynthesis-pathway?` to determine if a given pathway is biosynthetic or not. Other features will then help determine the specificity of a given biosynthetic pathway. Consider the case of *glycine biosynthesis III* pathway. In both E. coli and A. thaliana f_1 registers a 1 (true) in the feature vector for bionsynthesis. However, E. coli does not encode *glycine biosynthesis III* pathway and the remaining features register 0 (negative) indicating that this pathway is not present in the genome. In contrast, additional features specifying this pathway in A. thaliana indicate the presence of this pathway in this organismal genome. This represents a binary classification problem

as described in [1] which was solved using diverse machine learning methods. In this work, we solve a multi-label classification problem. Consider the following example based on same two organisms:

$$
\begin{array}{r}
\\
\textbf{A. thaliana}\\
\textbf{E. coli}\\
\vdots
\end{array}
\begin{array}{c}
\begin{matrix} f_1 & f_2 & f_3 & \cdots & f_{12,452} \end{matrix}\\
\begin{pmatrix}
6 & 0 & 11 & \cdots & 1\\
2 & 0 & 14 & \cdots & 0\\
\vdots & \vdots & \vdots & \vdots & \vdots
\end{pmatrix}
\end{array}
\longrightarrow
\begin{array}{c}
\begin{matrix} \text{TCA cycle I (prokaryotic)} & \text{glycine biosynthesis III} & \text{L-asparagine biosynthesis I} & \text{L-homocysteine biosynthesis} & \cdots \end{matrix}\\
\begin{pmatrix}
0 & 1 & 1 & 1 & \cdots\\
1 & 0 & 1 & 0 & \cdots\\
\vdots & \vdots & \vdots & \vdots & \ddots
\end{pmatrix}
\end{array}
$$

where each row in the left matrix is an organism (or multiple organisms) consisting of $12,452$ properties, extracted from 3650 enzymatic reactions (ECs). For example, f_1 encodes a numeric value indicating the abundance of `EC-1.1.1.1`. For E. coli, this feature has the value of 2 whereas for A. thaliana, f_1 is equal to 6. Since we have no information about pathways in a given sample, we had to re-design many features described in Dale and colleagues. For example, the feature `fraction-total-ec-act-in-biosynthesis-pathway` evaluates the total number of ECs that act in biosynthetic pathways to the total number of ECs found in a given instance. This features was re-designed from `biosynthesis-pathway?` feature in [1]. Given instances corresponding to ECs (left matrix), the goal ($\rightarrow$) is to predict a set of pathways for each instance which can be represented in a binary matrix on the right, where 1/0 asserts the presence/absence of a pathway, thereby, putting our work in the multi-label multiple outputs problem. For example, for E. coli, we are given enzymatic reactions and their properties and the target of mlLGPR is to identify a set of 307 true positive pathways, including *TCA cycle I (prokaryotic)*. Therefore, the problems discussed in this paper, although inspired by, are distinct from the work of Dale and colleagues. We describe the objective of our work in the Definitions and Problem Formulation section and make a stronger statement regarding features engineering based on the reviewers comment modifying the following sentences in the primary text:

- Line 70: **from** "mlLGPR uses logistic regression and feature vectors based on the work of Dale and colleagues..." **to** "mlLGPR uses logistic regression and feature vectors inspired by the work of Dale and colleagues..."
- Line 113: **from** "We consider five types of feature vectors based on the work of Dale and colleagues..." **to** "We consider five types of feature vectors inspired by the work of Dale and colleagues..."
- Line 413: **from** "With respect to features engineering, five feature sets were adapted from Dale and colleagues [1] to guide the learning process." **to** 'With respect to features engineering, five feature sets were re-designed from Dale and colleagues [1] to guide the learning process."

With regard to features analysis, it is a computationally intensive task to analyze each individual feature as examined in [1]. For an exhaustive analysis we would need to execute mlLGPR over 10000 times (12452 (#total features) $\times$ 2526 (#pathways)). Therefore, in the name of efficiency we performed ablation studies using group-based features. This was mentioned in the Features Selection Section (line 293):

> "A series of feature set "ablation" tests were conducted using Synset-2 as a training set in a reverse manner, starting with only reaction abundance features (AB), a fundamental feature set consisting of 3650 features and then successively aggregating additional feature sets while recording predictive performance on golden T1 datasets using the settings and metrics described in Section Experimental Setup. Because testing individual features is not practical, this form of aggregate testing provides a tractable method to identify the relative contribution of feature sets to pathway prediction performance."

However, based on the reviewer's comments, we went back and analyzed a subsample of features that is included in the Analysis of Features section in the supplementary file ("`experiment.pdf`").

C2 It seems that the training data is important in learning. The authors should describe in more detail how the training data Synset-1 and Synset-2 have been prepared. How many organisms were used for each training data?

R2 We provided details in the Synthetic Samples Generation section in the supplementary ("`experiment.pdf`") file. With regard to the process of generating samples, it is based on the statistical suggestion made by Shafiei and colleagues: "the presence of a pathway may follow the Poisson distribution" [2]. For mlLGPR, this approach aids in reducing redundant and irrelevant features. We recognized that we had not clearly indicated this in the manuscript, and we thank the reviewer pointer this out. Accordingly, we made the following changes:

- We removed (T1-T3) from Synthetic Dataset in Fig 2 and its caption is changed **to** "mlLGPR workflow. Datasets spanning the information hierarchy are used in feature engineering. The Synthetic dataset with features is split into training and test sets and used to train mlLGPR. Test data from the Gold Standard dataset (T1) with features and Synthetic dataset (T1-3) with features is used to evaluate mlLGPR performance prior to the application of mlLGPR on experimental datasets (T4) from different sources."
- Line 188: **from** "For training we used two synthetic datasets *Synset* 1 and *Synset* 2 constructed from a list of MetaCyc pathways representing T1-3 organismal PGDBs." **to** "For training we constructed two synthetic datasets *Synset* 1 and *Synset* 2 based on the Poisson distribution to subsample pathways, aligning with the previous work [2], from a list of MetaCyc pathways."

C3 It is less clear what the authors want to show in Table 3 by evaluating with or without AB, RE, or PP? As far as this reviewer can see, the performance seems quite comparable regardless of the choice of the feature categories, such as AB, RE, or PP. Instead of using (or not using) the entire category of features, it might be more interesting to show what specific features are important for the performance. This reviewer strongly suggests that the authors evaluate the effects of more specific features, for example, some specific features in AB categories.

R3 As indicated in **R1**, we performed ablation studies based on groups. We mentioned this in the Features Selection Section (line 293):

> "A series of feature set "ablation" tests were conducted using Synset-2 as a training set in a reverse manner, starting with only reaction abundance features (AB), a fundamental feature set consisting of 3650 features and then successively aggregating additional feature sets while recording predictive performance on golden T1 datasets using the settings and metrics described in Section Experimental Setup. Because testing individual features is not practical, this form of aggregate testing provides a tractable method to identify the relative contribution of feature sets to pathway prediction performance. "

However, based on the reviewer's comments, we included the Analysis of Features section in the supplementary file ("`experiment.pdf`") to analyze a subsample of features. With regard to the choice of categories of features, we emphasize that without RE or PE features, mlLGPR will neglect pathways that have low number or no enzymatic reactions. This is described in the Analysis of Features section in the supplementary file. We also replaced (−) symbols for models in Table 3 with (+) (e.g. mlLGPR-AB to mlLGPR+AB) to indicate the addition operation.

C4 It seems that the authors did not compare their performance with Dale et al.'s work [18]. Dale et al. already used diverse machine learning methods to infer metabolic pathways. It seems that the predictor used by Dale et al. is considerably good. Please show how much improvement in accuracy was made by this work compared with Dale et al.'s classifiers.

R4 See **R1** above. We were unable to run the code of Dale and colleagues on contemporary data sets and were therefore unable to make a direct comparison using the performance metrics included in the mlLGPR paper. Moreover, mlLGPR expands the nature of the problem to include both organismal and multi-organismal datasets, something not addressed by Dale and colleagues.

C5 Table 1 should be placed in landscape orientation. The domain information is hard to read.

R5 This is a good point. We adopted the reviewer's suggestion.

C6 What is the baseline method in Table 5?

R6 We explained the BASELINE method in the Experimental Setup section (line 204) of the main manuscript:

"In the baseline method, the enzymatic reactions of $\mathbf{x}^{(i)}$ for an instance i are mapped directly onto the true representation of all known pathways $\mathcal{Y}$."

However, we modified the above sentence to:

"In the BASELINE method, the enzymatic reactions of an example $\mathbf{x}^{(i)}$ are mapped directly onto the true representation of all known pathways $\mathcal{Y}$. Then, we apply a cutoff threshold (0.5) to retrieve a list of pathways for that example."

Reviewer B.

C1 It may not be evident to biologists as to what milGPR actually does. Metapathways has good descriptions of the approaches and graphical overviews of the approaches that may be useful to implement here as well. That being said, the previous paper by Dale et al. and Metapathways may not be obvious to everyone. I suggest adding a brief description of Metapathways and how mlLGPR builds upon it.

R1 We thank the reviewer for their insight on the potential relationship between MetaPathways and mlLGPR. In the current manuscript our primary goal was to explore the use of mlLGPR for pathway inference, and to benchmark the method as a prerequisite for potential integration into pipelines such as MetaPathways. Because of the need to first validate this approach we were hesitant to provide too much information regarding such an integration step. In future we anticipate developing a paper in which we make the integration process explicit and carefully explain how different prediction methods can be integrated within MetaPathways across the genomic information hierarchy.

C2 While there is sufficient description of what goes on under the hood – how this actually looks is not well described. What are typical inputs, what are typical results?

R2 Again a very pertinent point. It was our intention to provide in-depth guidelines on the GitHub page (mlLGPR). In particular, mlLGPR is a package that performs preprocessing from PGDBs, generates synthetic samples, trains pathway models, and predicts pathways. Inputs vary based on the preferred operations, but, the main goal is to provide a set of probable true pathways given a list of enzymatic reactions that are encoded in a clearly defined matrix format. However, based on the next comment we recognize that we can and should do better on the documentation side of things.

C3 I found the github page and readme to be very underwhelming. Additional details would be extremely useful. Two examples of confusion: 1. clone directory and download a bunch of databases (there are a couple of steps before this: Register with Pathway Tools, Databases were 300 Gb zipped, which we had to expand and then only keep six. This might be a serious limitation. Example: Bunch of arguments in parentheses (hard to understand what they are). I think clearer instruction on installation of dependencies would be useful. python3.5 pip -m install NumPy >= 1.15 etc. Bioinformatics is being made increasingly accessible and I find this to be necessary.

R3 The reviewer is spot on. We updated the "`README.md`" file with detailed elaborations about installations and guidelines to running mlLGPR. We cannot include PGDBs in github or any other repositories per guideline by SRI international (biocyc). However, we provided a file ("`object.pkl`") that includes the required information by mlLGPR. With regard to installation, we included "`requirement.txt`" file in the source folder.

C4 Another dependency that isn't mentioned is fuzzywuzzy, so please add this. python3.5 pip -m install fuzzywuzzy

R4 Again, we thank the reviewer for pointing out this omission. We included "fuzzywuzzy" in the "`requirement.txt`" file.

C5 The readme only contains a general command. Specifying the general arguments and describing each of them would be useful. What do they do, what are general ranges for their use?

> python main.py –biocyc –train –evaluate –predict –build_syn_dataset –nSample 15000 –average_item_per_sample 500 –build_synthetic_features –build_golden_dataset –build_golden_features –extract_info_mg –build_mg_features –ds_type ”syn_ds” –trained_model ”mlLGPR_en_ab_re_pe.pkl” –kbpath ”[MetaCyc location]” –dspath ”[Location to the processed dataset]” –mdpath ”[Location to store or save the model]” –rspath ”[Resuls location]” –ospath ”[Object location]” –n_jobs 10 –nEpochs 10 –nBatches 5

Therefore, I inferred there are 5 arguments to specify:

> –kbpath ”[MetaCyc location]” –dspath ”[Location to the processed dataset]” –mdpath ”[Location to store or save the model]” –rspath ”[Results location]” –ospath ”[Object location]”

From the help page:

> –ospath OSPATH The path to the data object that contains extracted information from the MetaCyc database. The default is set to object folder outside the source code.
>
> –rspath RSPATH The path to the results. The default is set to result folder outside the source code.
>
> –mdpath MDPATH The path to the output models. The default is set to train folder outside the source code.
>
> –dspath DSPATH The path to the dataset after the samples are processed. The default is set to dataset folder outside the source code.
>
> –kbpath KBPATH The path to the MetaCyc database. The default is set to database folder outside the source code.

R5 We updated the instructions for each command according to the reviewer's suggestions.

C6 In the github page there are 5 custom arguments, but from the help page, it is only required to make sure the kbpath argument is correct to run correctly. It would be informative to have a simpler example command to show the user how they can run the program with minimum amounts of setting changes.

R6 We now provide multiple examples for executing mlLGPR in the "`README.md`" file according to the reviewer's suggestions. We really this user-oriented feedback to help sustaining this open source tool.

C7 When giving the –ospath argument to be the folder with metacyc/data/, I get: the error message looks for a file named "object.pkl" in whichever folder I'm giving it. I am not sure what to make of this pickle file or where to look for it since there is nothing in the documentation.

R7 We noted the suggestion and subsequently uploaded several files to zenodo which are outlined in the "`README.md`" file.

C8 How fast is mlLGPR in comparison to other tools? Given that we had trouble installing and running it, we could not test run times.

R8 We thank the reviewer for this suggestion. We added the Run-Time Performance of Pathway Prediction Algorithms section in the supplementary file ("`experiment.pdf`") for analyzing time complexity using all pathway prediction algorithms. In summary, mlLGPR has the best overall prediction time in contrast to PathoLogic which has a less optimal prediction time.

C9 Fig 1B. This figure is unclear. What are the bars for SAGs much larger than for MAGs?

R9 The scale bars both sum to unity as a representation of genome completion. Single-cell amplified genomes (SAGs) are considered organismal in nature and therefore span T1-T3 levels of the genomic information hierarchy. The bar was extended to capture this relationship between tier and organismal identity and to reflect the fact that SAGs are typically span a wider range of completion states. Metagenome assembled genomes (MAGs) on the other hand are not organismal in nature and represent a population of closely related genomes grouped together based on statistical properties included k-mer frequency distribution, coverage and G+C content. Because they fall as a special case within the T4 category, which also includes metagenomic contigs, they are accorded a shorter completion bar.

C10 Figure 6: If pathways are not present as indicated by red circles, how is abundance information being shown?

R10 The red circles represent the presence of pathways which were not predicted by both PathoLogic and mlLGPR. This is stated in the caption of Figure 6:

"Red circles indicate that neither method predicted a specific pathway while green circles indicate that both methods predicted a specific pathway."

C11 Supplementary tables 1 and 2 are not easy to interpret. There are no captions. What do the numbers precisely indicate?

R11 We appreciate the comment, and we update the files and incorporated the following text in the caption of Supplementary tables 1 and 2, respectively:

"MetaCyc Pathway ID: The unique identifier for the pathway as provided by MetaCyc; MetaCyc Pathway Name: The name of the pathway as outlined by MetaCyc; Moranella: the Moranella endosymbiont (GenBank NC-015735); Tremblaya: the Tremblaya endosymbiont (GenBank NC-015736); Composite: a composite genome consisting of both endosymbiont genomes. Each numeric value encodes the coverage information of a pathway associated with each endosymbiont or composite genome. The coverage is computed based on mapping enzymes onto true representations of each pathway and is within the range of $[0, 1]$, where 1 indicates that all enzymes catalyzing reactions in a given pathway were identified while 0 means no enzymes were observed for a given pathway."

"MetaCyc Pathway ID: The unique identifier for the pathway as provided by MetaCyc; MetaCyc Pathway Name: The name of the pathway as outlined by MetaCyc; 25m: the 25 m depth interval corresponding in the HOTS water column; 75m: the 75m depth interval in the HOTS water column; 110m: the 110 m depth interval in the HOTS water column; and 500m: the 500 m depth interval in the HOTS water column. Each numeric value encodes abundance information for a given pathway associated with each depth interval. The abundance is expected pathway copies normalized based on mapping enzymes onto true representation of each pathways."

C12 Spelling: Metabaolic in abstract.

R12 We thank the reviewer for highlighting the misspelling of the word and we correct it accordingly.

References

[1] Joseph M Dale, Liviu Popescu, and Peter D Karp. Machine learning methods for metabolic pathway prediction. *BMC bioinformatics*, 11(1):1, 2010.

[2] Mahdi Shafiei, Katherine A Dunn, Hugh Chipman, Hong Gu, and Joseph P Bielawski. Biomenet: A bayesian model for inference of metabolic divergence among microbial communities. *PLoS Comput Biol*, 10(11):e1003918, 2014.
