# Supplementary material for: Metabolic pathway inference using multi-label classification with rich pathway features
Source: PLoS Comput Biol. 2020 Oct 1;16(10):e1008174. doi: 10.1371/journal.pcbi.1008174 (PMC7529316; doi:10.1371/journal.pcbi.1008174)
Supplement: Attachment — Submitted filename: Basher et al. PLOS Comp Bio 04.06.2020.docx [file pcbi.1008174.s006.docx]

This paper by Basher et al. describes an approach to perform pathway prediction using multi-label classification in lieu of gene-centric approaches. The study tackles an important problem – inference of metabolic pathway predictions in ever-increasing complex datasets and builds on prior work from the authors, including development of Metapathways. This is a good paper with significant potential to make an important contribution to the field. The approaches used are mostly solid and math described, tests conducted, and benchmarking is robust. Results indicate that milGPR performs well in comparison to other tools. However, I do have significant concerns with some of the description and details provided. As currently described, the software is extremely difficult to run and derive results from. Below, I provide specific examples of issues and provide means to improve upon them.

**Major concerns**

It may not be evident to biologists as to what milGPR actually does. Metapathways has good descriptions of the approaches and graphical overviews of the approaches that may be useful to implement here as well. That being said, the previous paper by Dale et al. and Metapathways may not be obvious to everyone. I suggest adding a brief description of Metapathways and how milGPR builds upon it.

While there is sufficient description of what goes on under the hood – how this actually looks is not well described. What are typical inputs, what are typical results?

I found the github page and readme to be very underwhelming. Additional details would be extremely useful. Two examples of confusion: 1. clone directory and download a bunch of databases (there are a couple of steps before this: Register with Pathway Tools, Databases were 300 Gb zipped, which we had to expand and then only keep six. This might be a serious limitation.

Example: Bunch of arguments in parentheses (hard to understand what they are).

I think clearer instruction on installation of dependencies would be useful.

python3.5 pip -m install NumPy>=1.15 etc. Bioinformatics is being made increasingly accessible and I find this to be necessary

another dependency that isn't mentioned is fuzzywuzzy, so please add this.

python3.5 pip -m install fuzzywuzzy

The readme only contains a general command. Specifying the general arguments and describing each of them would be useful. What do they do, what are general ranges for their use?

python main.py --biocyc --train --evaluate --predict --build_syn_dataset --nSample 15000 --average_item_per_sample 500 --build_synthetic_features --build_golden_dataset --build_golden_features --extract_info_mg --build_mg_features --ds_type "syn_ds" --trained_model "mlLGPR_en_ab_re_pe.pkl" --kbpath "[MetaCyc location]" --dspath "[Location to the processed dataset]" --mdpath "[Location to store or save the model]" --rspath "[Resuls location]" --ospath "[Object location]" --n_jobs 10 --nEpochs 10 --nBatches 5

Therefore, I inferred there are 5 arguments to specify:

--kbpath "[MetaCyc location]"

--dspath "[Location to the processed dataset]"

--mdpath "[Location to store or save the model]"

--rspath "[Results location]"

--ospath "[Object location]"

From the help page:

--ospath OSPATH	The path to the data object that contains extracted

information from the MetaCyc database. The default is

set to object folder outside the source code.

--rspath RSPATH	The path to the results. The default is set to result

folder outside the source code.

--mdpath MDPATH	The path to the output models. The default is set to

train folder outside the source code.

--dspath DSPATH	The path to the dataset after the samples are

processed. The default is set to dataset folder

outside the source code.

--kbpath KBPATH	The path to the MetaCyc database. The default is set

to database folder outside the source code.

In the github page there are 5 custom arguments, but from the help page, it is only required to make sure the kbpath argument is correct to run correctly.

It would be informative to have a simpler example command to show the user how they can run the program with minimum amounts of setting changes.

When giving the --ospath argument to be the folder with metacyc/data/, I get:

the error message looks for a file named "object.pkl" in whichever folder I'm giving it. I am not sure what to make of this pickle file or where to look for it since there is nothing in the documentation.

How fast is milGPR in comparison to other tools? Given that we had trouble installing and running it, we could not test run times.

Fig 1B. This figure is unclear. What are the bars for SAGs much larger than for MAGs?

Figure 6: If pathways are not present as indicated by red circles, how is abundance information being shown?

Supplementary tables 1 and 2 are not easy to interpret. There are no captions. What do the numbers precisely indicate?

Spelling: Metabaolic in abstract
